# Conditional knockdown of hyaluronidase 2 in articular cartilage stimulates osteoarthritic progression in a mice model

**DOI:** 10.1038/s41598-017-07376-5

**Published:** 2017-08-01

**Authors:** Yoshitoshi Higuchi, Yoshihiro Nishida, Eiji Kozawa, Lisheng Zhuo, Eisuke Arai, Shunsuke Hamada, Daigo Morita, Kunihiro Ikuta, Koji Kimata, Takahiro Ushida, Naoki Ishiguro

**Affiliations:** 1 Department of Orthopedic Surgery, Nagoya University Graduate School of Medicine, Nagoya, Japan; 2 Multidisciplinary Pain Center, Aichi Medical University, Nagakute, Japan

## Abstract

The catabolism of hyaluronan in articular cartilage remains unclear. The aims of this study were to investigate the effects of hyaluronidase 2 (*Hyal2*) knockdown in articular cartilage on the development of osteoarthritis (OA) using genetic manipulated mice. Destabilization of the medial meniscus (DMM) model of Col2a promoter specific conditional *Hyal2* knockout (*Hyal* ^*−/−*^) mice was established and examined. Age related and DMM induced alterations of articular cartilage of knee joint were evaluated with modified Mankin score and immunohistochemical staining of MMP-13, ADAMTS-5, KIAA11199, and biotinylated- hyaluronan binding protein staining in addition to histomorphometrical analyses. Effects of *Hyal2* suppression were also analyzed using explant culture of an IL-1α induced articular cartilage degradation model. The amount and size of hyaluronan in articular cartilage were higher in *Hyal2* ^*−/−*^ mice. *Hyal2* ^*−/−*^ mice exhibited aggravated cartilage degradation in age-related and DMM induced mice. MMP-13 and ADAMTS-5 positive chondrocytes were significantly higher in *Hyal2* ^*−/−*^ mice. Articular cartilage was more degraded in explant cultures obtained from *Hyal2* ^*−/−*^ mice. Knockdown of *Hyal2* in articular cartilage induced OA development and progression possibly mediated by an imbalance of HA metabolism. This suggests that Hyal2 knockdown exhibits mucopolysaccharidosis-like OA change in articular cartilage similar to Hyal1 knockdown.

## Introduction

Hyaluronan (HA) is a major constituent of extracellular matrices (ECMs) of various tissues and belongs to the glycosaminoglycan (GAG) family of polysaccharides. Its structure is a nonsulfated GAG composed of repeating disaccharides; beta-(1,3)-linked-D-glucuronic acid and beta-(1,4)-linked-N-acetyl-D-glucosamine^1–5^. HA is abundant particularly in connective tissues including articular cartilage, synovial fluid of the joint and the vitreous humor of the eye^6^, and the turnover rate of HA is high in most connective tissues^7^. The balance of anabolism and catabolism in HA is crucial for maintenance of connective tissue quality. Synthesis of HA in articular cartilage, is regulated mainly by hyaluronan synthase 2 (HAS2) in man, and effects of anabolic or catabolic cytokines on HA synthesis and HAS mRNA expression have been revealed in previous studies^8–10^. On the other hand, the catabolic activity of crucial enzymes for HA, particularly in articular cartilage has not been clarified yet.

There are six human hyaluronidase-like genes that are localized in two chromosomal regions, 3p21.3 (*HYAL1, HYAL2, HYAL3*), and 7q31.3 (*HYAL4, PH-20, and HYALP1)* ^2^. The tissue-specific, restricted expression patterns of HYAL4, PH-20, and HYALP1 have been reported^11^, with HYAL1, HYAL2, and HYAL3 seeming to play critical roles in the degradation of HA.

Among the three putative active hyaluronidases, the ability to degrade HA by HYAL3 is questionable^12^. Therefore, two hyaluronidases (HYAL1 and HYAL2) and cell surface HA receptor (CD44) have been surmised to play key roles in HA degradation^2, 5^. Chow *et al*. demonstrated HYAL2 to be the predominant mRNA transcript expressed in articular chondrocytes^13^. Csoka *et al*. suggested collaboration between HYAL1 with HYAL2 to degrade HA^11^. Initial cleavage of HA might occur at the cell surface or within the acidic endocytic vesicles by HYAL2^14^. Intermediate size of cleaved HA is transported to the lysosomes for further degradation by HYAL1^15^.

A previous report analyzed only the effects of deficiency of *HYAL1*, not *HYAL2* on the development of osteoarthritis^16^, because knockdown of *Hyal2* in mice is associated with cardiopulmonary dysfunction and pre-weaning lethality^3, 4^.

Deficiency of *Hyal1* causes mucopolysaccharidosis (MPS) IX, a lysosomal storage disorder characterized by joint abnormalities due to HA accumulation and osteoarthritis^16^. In contrast, *Hyal3* knockout mice exhibited no gross phenotypic changes, nor histologic abnormalities of knee joints^17^. *Hyal2* conditional knockout mice were non-lethal, and displayed craniofacial and cervical vertebral abnormalities, but the effect of this mutation on articular cartilage and development of osteoarthritis has not been reported^3^.

The aims of the current study were to analyze the roles of Hyal2 in the development and progression of osteoarthritis using non-treated aged mice (natural course) and joint instability OA models of mice, in addition to an explant culture inflammation model of articular cartilage. To achieve these aims, we generated Col2a1-dependent conditional knockout mice using the Cre-loxP system focusing on the *Hyal2* deficiency in articular cartilage.

## Results

### Natural course of conditional *Hyal2*^*−/−*^ mice

Macroscopically, *Hyal2* ^*+/Flox*^, *Hyal2* ^*+/*^ and *Hyal2* ^*−/−*^ neonates exhibited no obvious gross phenotypic abnormalities. Soft X-ray analyses of 3- and 9-month-old mice revealed no significant differences in skeletal formation between *Hyal2* ^*−/−*^ and WT mice. Although tibia bone length of *Hyal2* ^*−/−*^ mice at 9 months was slightly shorter than that of WT, the difference is statistically significant (*Hyal2*:^*−/−*^ 17.5 ± 0.2 mm, WT: 17.9 ± 0.1mm, P < 0.001). There was no statistical difference between the body weight of *Hyal2* ^*−/−*^ and WT mice at 9 months of age (*Hyal2*:^*−/−*^ 34.4 ± 3.5 g, WT: 32.4 ± 3.5).

**Figure 1:**
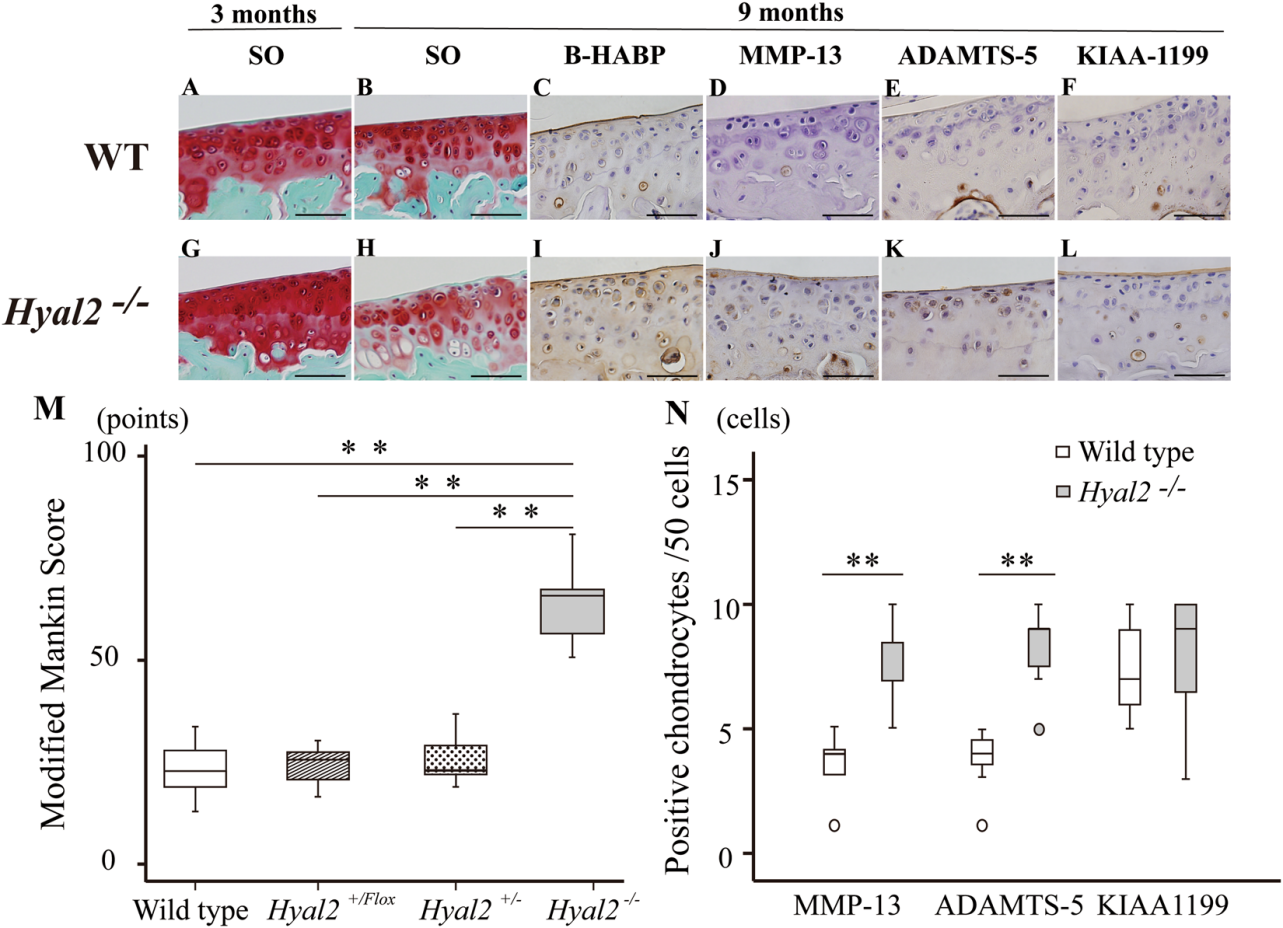
Histologic analysis of structural damage of articular cartilage in natural course of WT and *Hyal2* ^*−/−*^ mice at 3 and 9 months of age. Safranin-O staining (SO): WT and *Hyal2* ^*−/−*^ mice at 3 months (A and G, respectively) and 9 months (B and H, respectively). B-HABP staining: WT and *Hyal2* ^*−/−*^ mice at 9 months (C and I, respectively). Immunohistochemical staining for MMP-13: WT and *Hyal2* ^*−/−*^ mice at 9 months (D and J, respectively). Immunohistochemical staining for ADAMTS-5: WT and *Hyal2* ^*−/−*^ mice at 9 months (E and K, respectively). Immunohistochemical staining for KIAA1199: WT and *Hyal2* ^*−/−*^ mice at 9 months (F and L, respectively). Scale bars depict 50 μm in length. Modified Mankin score of medial tibia in WT and *Hyal2* ^*−/−*^ mice at 9 months (**P < 0.01) (M). The numbers of immunostaining positive chondrocytes for MMP-13, and ADAMTS-5 in *Hyal2* ^*−/−*^ (K/O) mice were significantly elevated compared to that in WT mice (**P < 0.01, respectively) (N). The number of positive chondrocytes for KIAA1199 in the articular cartilage was not statistically different between the two groups. Small circle represented outlier.

Results of Safranin-O staining demonstrated that there was no significant difference in stainable proteoglycan of articular cartilage between *Hyal2* ^*−/−*^ and WT mice at 3 months of age (Fig. 1A, G). However, at 9 months, there was a marked reduction in *Hyal2* ^*−/−*^ mice compared with WT mice (Fig. 1H and B, respectively). Total modified Mankin score in *Hyal2* ^*−/−*^ was significantly higher than that in WT, *Hyal2* ^*+/Flox*^, *Hyal2* ^*+/*^ mice at 9 months (P < 0.01, Fig. 1M). Modified Mankin score indicated that only minimal OA change occurred in WT mice, similar to that in *Hyal2* ^*+/Flox*^, *Hyal2* ^*+/*^ mice at 9 months. It should be noted that the growth plate physiology differs between humans and mice, since the growth plates do not close directly after sexual maturation in mice. The growth plate heights were slightly reduced in *Hyal2* ^*−/−*^ mice compared with those in WT mice at 9 months. In comparison with the 3-month-old ones, the heights in both 9-month-old *Hyal2* ^*−/−*^ and WT mice were reduced probably due to their sexual maturation (Supplementary Fig. S1). HABP positivity in the growth plate was higher in *Hyal2* ^*−/−*^ mice compared with WT mice. HABP staining revealed a difference in HA accumulation in articular cartilage between WT and *Hyal2* ^*−/−*^ mice (Fig. 1C and I, respectively).

MMP-13 positive chondrocytes were observed in the area where safranin O staining was decreased in 9-month-old *Hyal2* ^*−/−*^ mice (Fig. 1J). In contrast, fewer MMP-13 positive chondrocytes were observed in articular cartilage of WT mice (Fig. 1D), and statistically significant (P < 0.01, Fig. 1N). More ADAMTS-5-positive chondrocytes were observed in *Hyal2* ^*−/−*^ articular cartilage compared with those in WT cartilage (Fig. 1K and E, respectively) (P < 0.01, Fig. 1N). There was no significant difference in the ratio of articular chondrocytes positive for KIAA1199 between the two groups (*Hyal2*:^*−/−*^ Fig. 1L, WT: Fig. 1F, P > 0.05: Fig. 1N). Micro CT did not show significant differences between the two groups (Supplementary Table S1).

**Figure 2:**
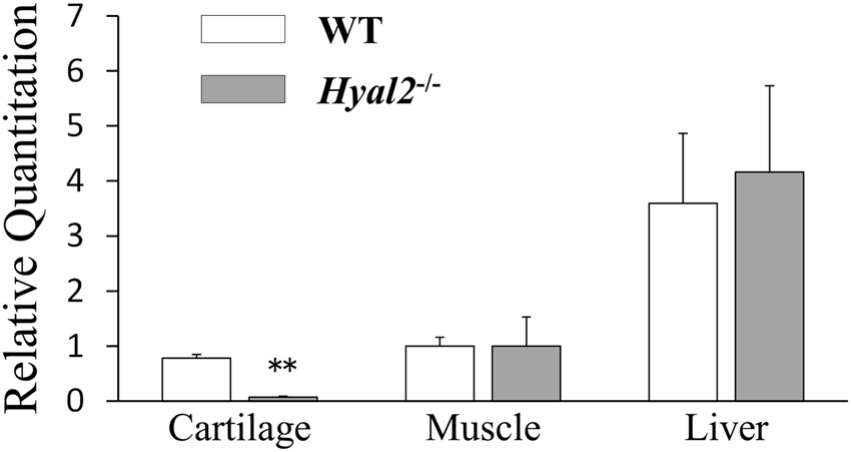
Hyal2 mRNA expression in cartilage, muscle and liver of 9-month-old male *Hyal2* ^*−/−*^ and WT mice. Expression levels of Hyal2 mRNA were determined with RT-PCR, and normalized with those of Gapdh. (n = 6, respectively). **P < 0.01.

**Figure 3:**
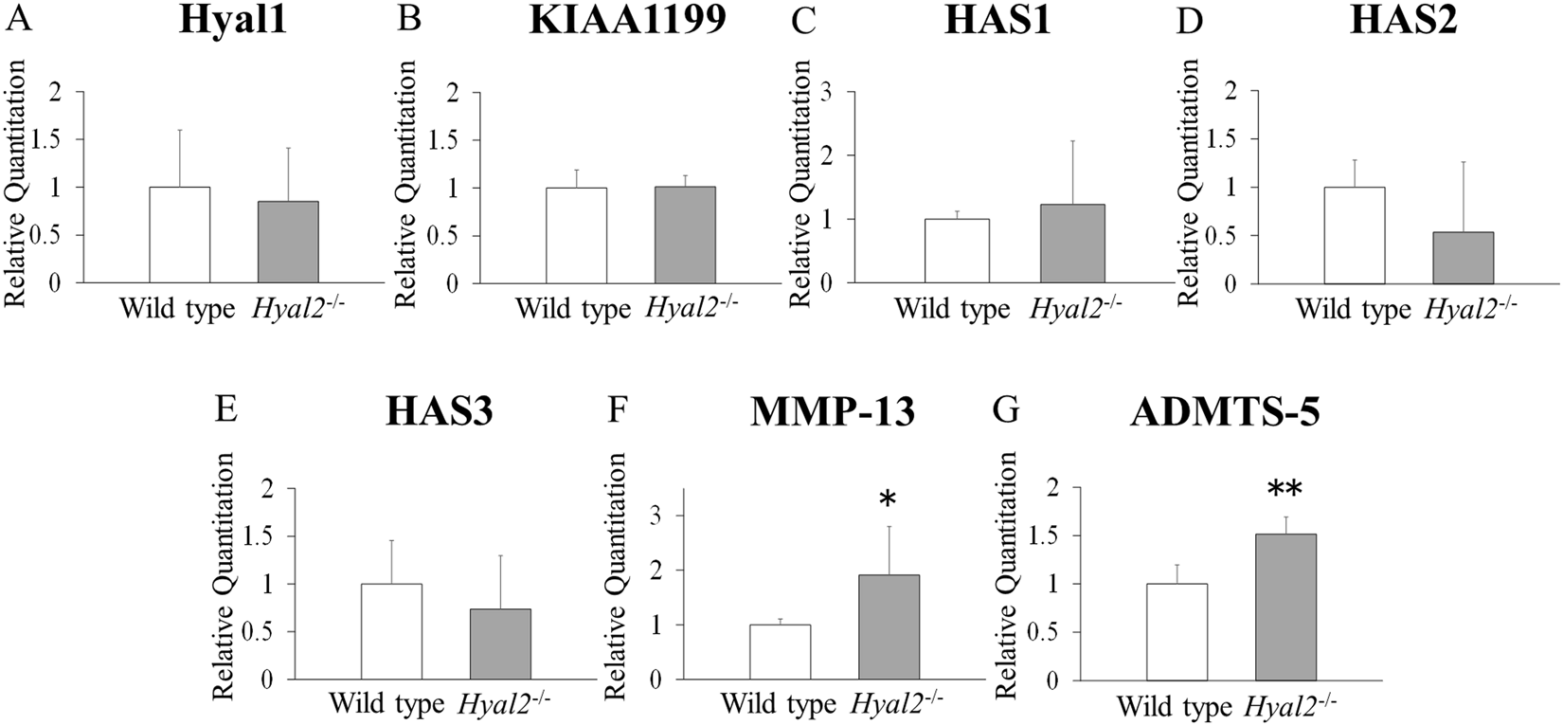
mRNA expression levels of hyaluronan-related molecules and proteolytic enzyme of extracellular matrix. mRNA expression of Hyal1(**A**), KIAA1199(**B**), HAS1(**C**), HAS2(**D**), HAS3(**E**), MMP-13(**F**), ADAMTS-5(**G**) in articular cartilage from 9-month-old male *Hyal2* ^*−/−*^ and WT mice as determined by real-time PCR amplification (n = 6, respectively). The data presented are the average ± S.D of relative mRNA expression values standardized by Gapdh mRNA expression. *P < 0.05, **P < 0.01.

Results of RT-PCR analyses revealed that Hyal2 mRNA expression was specifically reduced in articular cartilage of *Hyal2* ^*−/−*^ mice compared with that of WT mice (Fig. 2). There was no significant difference in mRNA expression of Hyal1, KIAA1199, and HAS1–3 between *Hyal2* ^*−/−*^ and WT mice (Fig. 3A–E). On the other hand, expression levels of MMP-13, ADAMTS-5 mRNA were significantly increased in articular cartilage of *Hyal2* ^*−/−*^ mice compared with that of WT mice (Fig. 3F and G, respectively), which was consistent with the results of immunohistochemistry.

### Effect of *Hyal2* deficiency in the amount and size of cartilage hyaluronan

The obtained glycosaminoglycans were subjected to HA quantification using competitive ELISA, which is more sensitive for low molecular weight HA than sandwich ELISA^18^. The yields of joint HA from *Hyal2* ^*−/−*^ and WT mice were 55.3 ± 9.7 and 33.7 ± 17.5 ng/mg wet joint, respectively. Obviously, *Hyal2* deficiency has significantly caused the accumulation of hyaluronan in joint cartilage (p = 0.025, n = 6).

**Figure 4:**
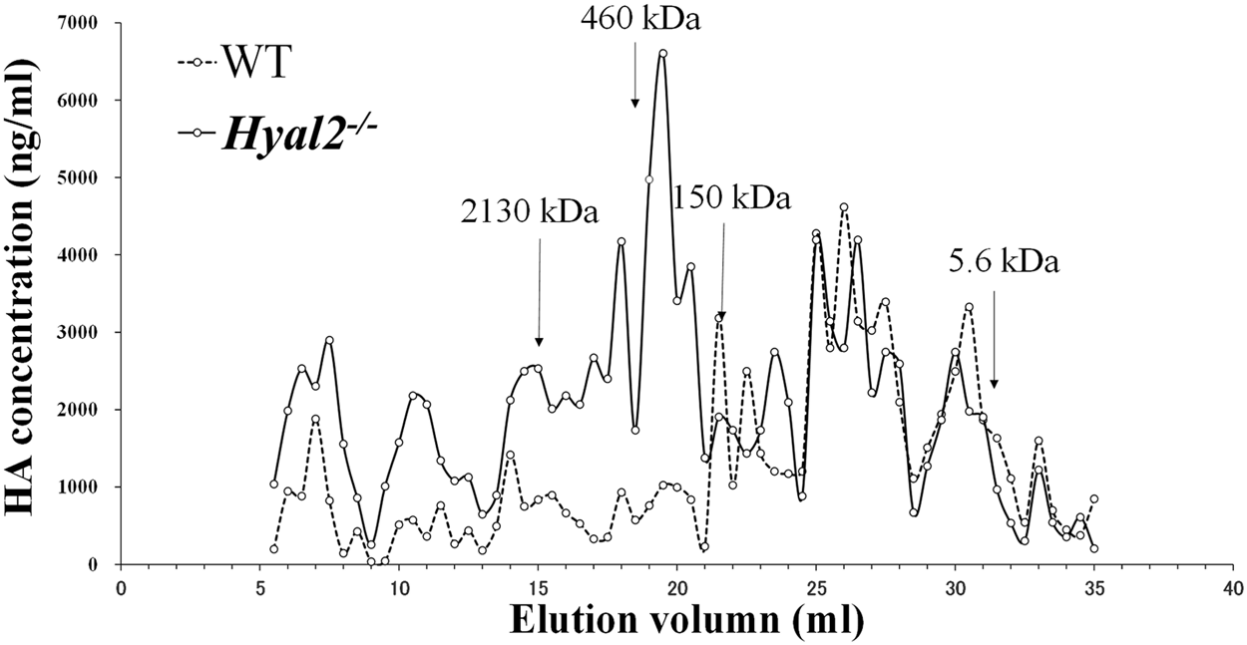
Hyal2 deficiency in cartilage causes the accumulation of high molecular weight hyaluronan. Hyaluronan extracted from knee joints and femoral heads of 9-month-old male mice and hyaluronan references with known molecular sizes were subjected to Sephacryl S-1000 gel filtration chromatography (0.7 × 40 cm). Hyaluronan contents in each fraction were determined using a competitive ELISA. The arrows indicate the positions of peaks of hyaluronan references.

We then further examined whether the molecular size of HA also altered. The glycosaminoglycan preparations from 6 mice were pooled and subjected to Sephacryl S-1000 gel filtration chromatography. As shown in Fig. 4, HA molecules with a molecular mass bigger than 280 kDa are greatly increased in *Hyal2* ^*−/−*^ mice, while the low molecular weight HA molecules are comparable between *Hyal2* ^*−/−*^ and WT mice.

Collectively, the data clearly indicate a role of Hyal2 in the turnover of cartilage HA. In *Hyal2* ^*−/−*^ mice, HA content increases by about 60%, most of which are high molecular weight molecules with molecular masses bigger than 280 kDa.

### OA development in mice subjected to destabilization of the medial meniscus (DMM) surgery

There was no significant difference between the body weight of *Hyal2* ^*−/−*^ and WT mice at 6 or 8 weeks after DMM surgery. (*Hyal2*:^*−/−*^ 29.8 ± 2.8 g and 31.6 ± 2.4 g, WT: 28.0 ± 1.1 g and 29.6 ± 1.4 g, respectively. P = 0.144 and 0.072) However, at 10 weeks after surgery, the average body weight of *Hyal2* ^*−/−*^ mice was greater than that of WT mice (*Hyal2*:^*−/−*^ 32.3 ± 2.4 g; WT: 28.6 ± 1.2 g, p < 0.05)

**Figure 5:**
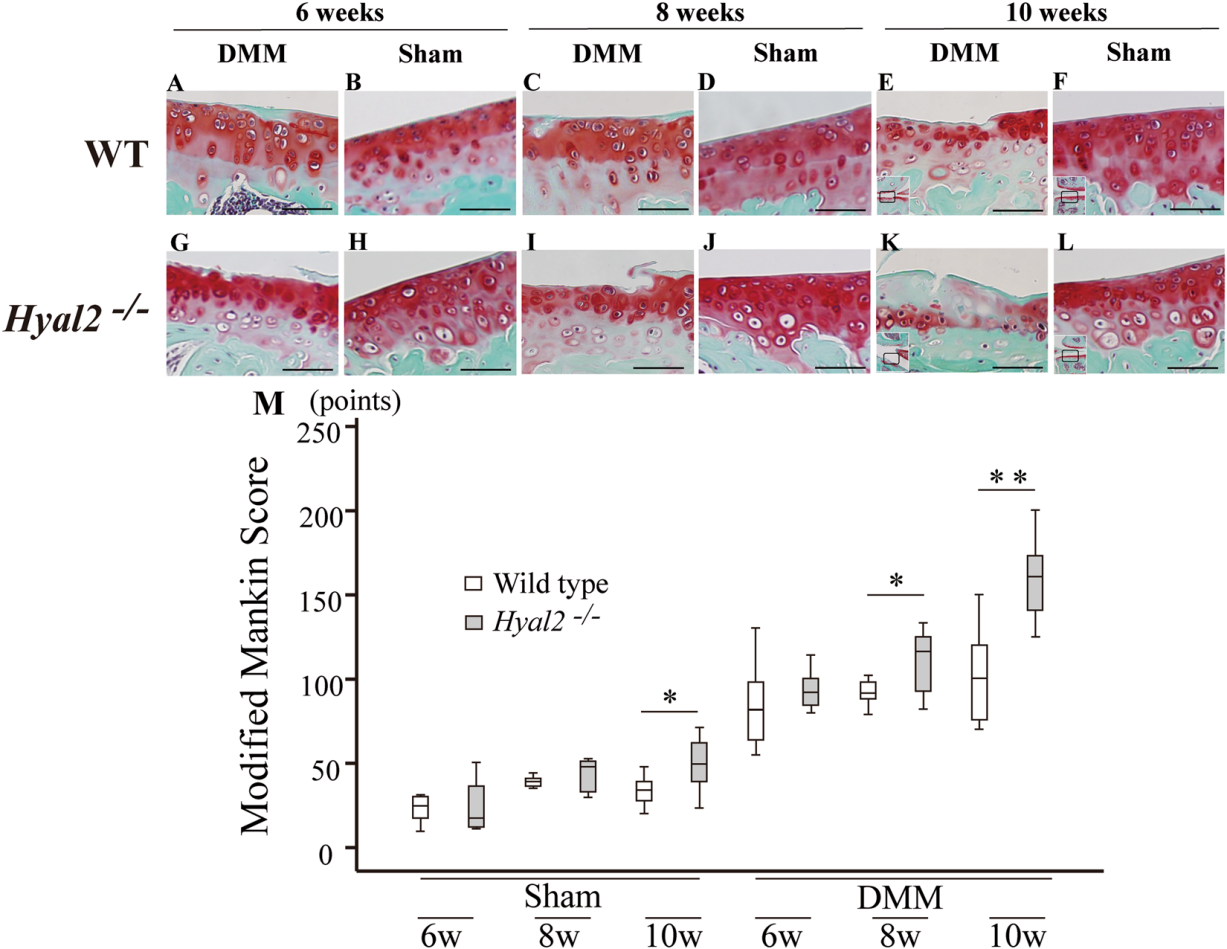
Histological findings of articular cartilage in DMM and sham treated WT and *Hyal2* ^*−/−*^ mice. Safranin O staining of articular cartilage in sham treated (B, D, F, H, J, L) and DMM treated (A, C, E, G, I, K) mice. Bars depict 50μm in length. Modified Mankin score (M) of mice articular cartilage (n = 8 per group). * P < 0.05, ** P < 0.01.

**Figure 6:**
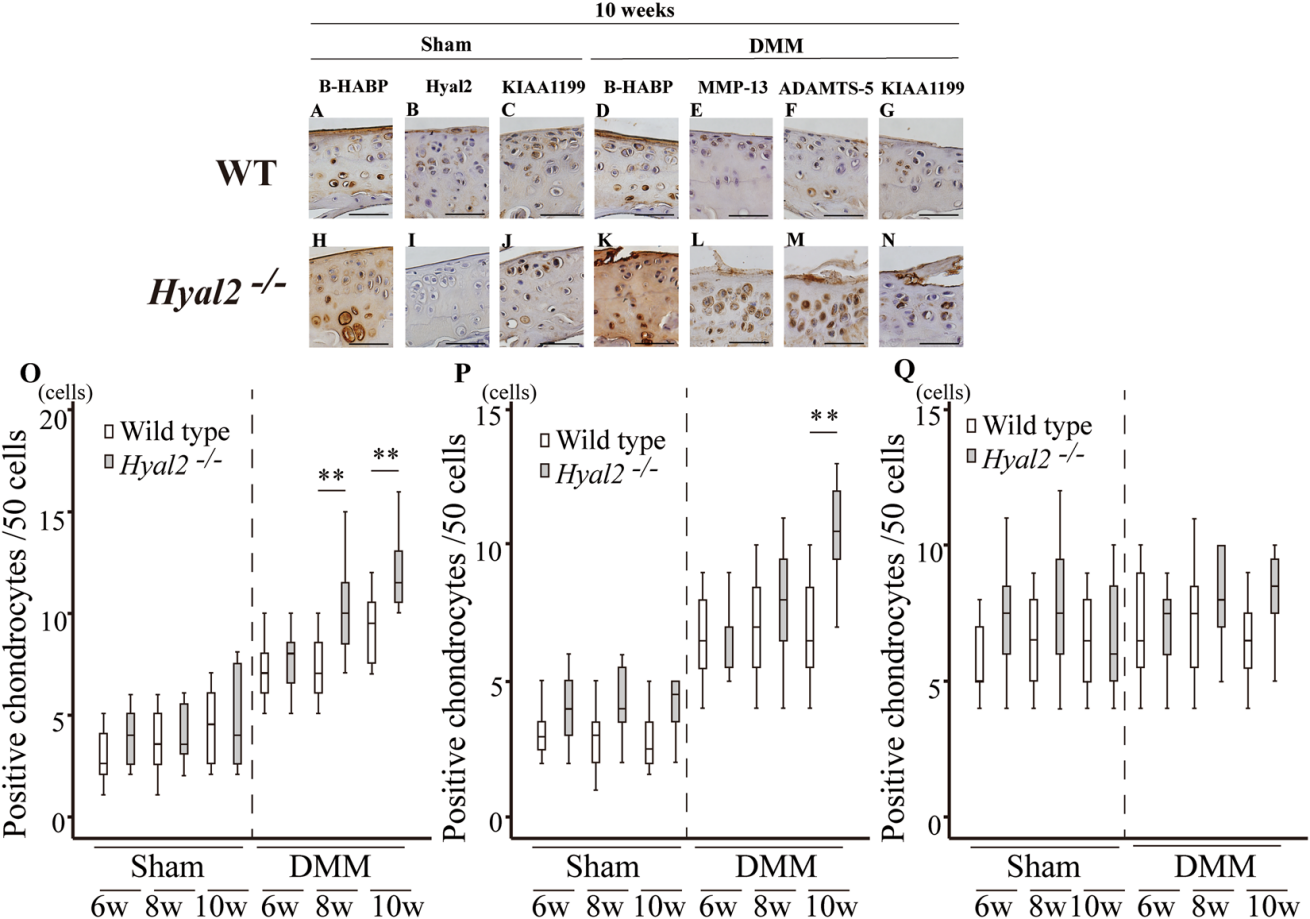
Results of b-HABP and immunohistochemical staining. Staining of articular cartilage in medial tibia at 10 weeks after surgery. B-HABP staining of sham operated side (A; WT, H; *Hyal2* ^*−/−*^) and DMM operated side (D; WT, K; *Hyal2* ^*−/−*^). Hyal2 (B; WT sham, I; *Hyal2* ^*−/−*^ sham), MMP-13 (E; WT DMM, L; *Hyal2* ^*−/−*^ DMM), ADAMTS-5 (F; WT DMM, M; *Hyal2* ^*−/−*^ DMM), and KIAA1199 (C; WT sham, J; *Hyal2* ^*−/−*^ sham, G; WT DMM, N; *Hyal2* ^*−/−*^ DMM) at 10 weeks after surgery. The numbers of positive chondrocytes for MMP-13 (O), ADAMTS-5 (P), and KIAA1199 (Q) were graphed (** P < 0.01).

As for differences between sham-treated *Hyal2* ^*−/−*^ and WT, Safranin O staining revealed no marked reduction of stainable proteoglycan in articular cartilage of either sham-treated *Hyal2* ^*−/−*^ or WT mice at 6, 8, or 10 weeks after surgery (Fig. 5B and H, D and J, F and L, respectively). There were no significant differences in modified Mankin scores at 6 or 8 weeks between *Hyal2* ^*−/−*^ (sham) and WT (sham) mice. In contrast, the difference was significant at 10 weeks (P = 0.038) (Fig. 5M). As expected, the results of HABP staining revealed greater accumulation of HA in articular cartilage of *Hyal2* ^*−/−*^ mice (sham) (Fig. 6H) compared with WT (sham) (Fig. 6A). Interestingly, lacunae of the deeper cells of articular cartilage of the *Hyal2* ^*−/−*^ mice (sham) (Fig. 5H,J and L) were so large and odd-looking compared to those of WT mice (sham) (Fig. 5B,D and F). Considering that these large lacunae exhibited strong positive staining for HABP (Fig. 6H), suggesting the HA accumulation by *Hyal2* knockout might cause the enlargement of lacunae, particularly of deeper cells.

Dark staining of HA was observed in and around the chondrocytes in addition to the territorial area of ECM of *Hyal2* ^*−/−*^sham (Fig. 6H). There was no difference in the staining positivity for KIAA1199 between *Hyal2* ^*−/−*^ (sham) (Fig. 6J) and WT mice (sham) (Fig. 6C). Micro CT analysis at 6 weeks after surgery showed no significant difference in any of the parameters between *Hyal2* ^*−/−*^ (sham) and WT (sham) mice (Supplementary Table S2), whereas, at 8 weeks, subchondral bone plate thickness (SbTh) and trabecular thickness (TbTh) in *Hyal2* ^*−/−*^ (43.0 ± 4.1 and 54.6 ± 5.3 μm, respectively) were significantly lower than those in WT (SbTh: 48.5 ± 5.2, TbTh: 61.6 ± 6.0 μm). At 10 weeks after sham surgery, almost all parameters in *Hyal2* ^*−/−*^ were significantly lower than those in WT (Table 1). The differences noted in the parameters of bone morphometry might be partly attributable to abnormal HA accumulation during endochondral bone formation, particularly in the growth plate.

**Table 1:** Micro CT analysis at 10 weeks after DMM surgery.

Parameter	WT DMM (n = 8)	WT Sham (n = 8)	*Hyal2* ^*−/−*^ DMM (n = 8)	*Hyal2* ^*−/−*^ Sham (n = 8)	WT relative change (%)	*Hyal2* ^*−/−*^ relative change (%)
Sb BV/TV (%)	57.2 ± 8.6*	44.1 ± 7.5	52.0 ± 5.5†	32.7 ± 10.2‡	25.1 ± 4.0	59.9 ± 14.0§
Sb Th (μm)	60.4 ± 3.1*	52.5 ± 3.5	51.3 ± 5.7†¶	42.9 ± 7.4‡	12.8 ± 7.0	24.1 ± 8.7
Tb BV/TV (%)	41.1 ± 4.2	43.7 ± 2.7	30.5 ± 5.8¶	31.3 ± 7.6‡	−5.5 ± 7.0	−2.3 ± 2.6
Tb Th (μm)	58.2 ± 2.7	61.0 ± 6.6	50.1 ± 6.5¶	52.0 ± 3.6‡	−3.8 ± 4.6	−4.9 ± 3.6
Tb Sp (μm)	101.6 ± 18.0*	85.2 ± 5.4	119.9 ± 14.0†¶	99.3 ± 12.7‡	19.3 ± 11.2	17.9 ± 4.0
Tb.N (/mm)	7.1 ± 0.8	7.9 ± 0.6	6.1 ± 1.0¶	6.8 ± 1.6‡	−9.8 ± 8.2	−11.1 ± 6.4

Relative increase of parameters was calculated as follows [(value of DMM – value of Sham) ÷ value of sham × 100%]

*Statistically significant difference between Wild DMM and Wild Sham.

†Statistically significant difference between *Hyal2* ^*−/−*^ DMM and *Hyal2* ^*−/−*^ Sham.

‡Statistically significant difference between *Hyal2* ^*−/−*^ Sham and Wild Sham.

§Statistically significant difference between relative increase *Hyal2* ^*−/−*^ and Wild.

¶Statistically significant difference between *Hyal2* ^*−/−*^ DMM and Wild DMM.

Articular cartilage in DMM joints showed a significantly higher modified Mankin score than that in Sham joints (Fig. 5M). These results confirmed the adequacy of the DMM model used in the present study. Regarding the difference between DMM surgery of *Hyal2* ^*−/−*^ and WT cartilage, the results of Safranin O staining at 10 weeks revealed that stainable proteoglycan was reduced in *Hyal2* ^*−/−*^ (DMM) mice compared to WT (DMM) mice (Fig. 5K and E, respectively). At 8 and 10 weeks after DMM surgery, articular cartilage in *Hyal2* ^*−/−*^ (Fig. 5I and K) mice showed osteoarthritic structural change as compared with WT mice (Fig. 5C and E).

HA deposition detected by HABP staining was increased in the pericellular area and in and around the chondrocytes in *Hyal2* ^*−/−*^ mice compared with WT mice with DMM surgery at 10 weeks (Fig. 6K and D, respectively). More MMP-13 positive (Fig. 6L) and ADAMTS-5 positive chondrocytes (Fig. 6M) were observed in *Hyal2* ^*−/−*^ mice (DMM) at 10 weeks than in WT mice (Fig. 6E and F, respectively). HA deposition in *Hyal2* ^*−/−*^ mice was also increased compared with WT mice with DMM surgery at 6 and 8 weeks. MMP-13 positive chondrocytes were significantly more observed *Hyal2* ^*−/−*^ mice compared with WT mice with DMM surgery at 8 and 10 weeks (P < 0.01, Fig. 6O), while there was no difference at 6 weeks. The number of ADAMTS-5 positive chondrocytes was significantly higher in articular cartilage of *Hyal2* ^*−/−*^ (DMM) mice at 10 weeks than in WT (DMM) mice (P < 0.01, Fig. 6P), while there was no statistical difference at 6 or 8 weeks. The number of KIAA1199 positive chondrocytes (Fig. 6G and N) was not statistically different between the two groups (*Hyal2* ^*−/−*^ with DMM vs WT with DMM) (Fig. 6Q).

Micro CT analysis did not show any significant differences in the bone morphometric parameters between *Hyal2* ^*−/−*^ and WT DMM mice at 6 weeks after surgery (Supplementary Table S2). SbTh and TbTh at 8 and 10 (Table 1) weeks were lower in *Hyal2* ^*−/−*^ than in WT (*Hyal2* ^*−/−*^ DMM vs WT DMM; p < 0.05) (*Hyal2* ^*−/−*^ Sham vs WT Sham; p < 0.05). In order to analyze the effects of *Hyal2* knockout with or without DMM surgery on the changes in morphometric parameters, relative changes in parameters from sham to DMM was were calculated as previously described^19^. Relative change in the parameters was calculated as follows: [(“value of DMM” minus “value of sham”) / “value of sham”] × 100. The relative increase of SbTh and TbTh at 8 weeks was not significantly different between *Hyal2* ^*−/−*^ and WT mice. Scores of Sb BV/TV, SbTh and TbSp of both *Hyal2* ^*−/−*^ and WT were significantly higher in joints with DMM than those with the sham procedure at 10 weeks after surgery (Table 1). Relative change of Sb BV/TV was significantly increased in *Hyal2* ^*−/−*^ DMM as compared with WT DMM (P < 0.05), indicating the *Hyal2* knockout accelerates the sclerotic change of subchondral bone.

### Effects of IL-1α on *Hyal2* deficient cartilage explant

**Figure 7:**
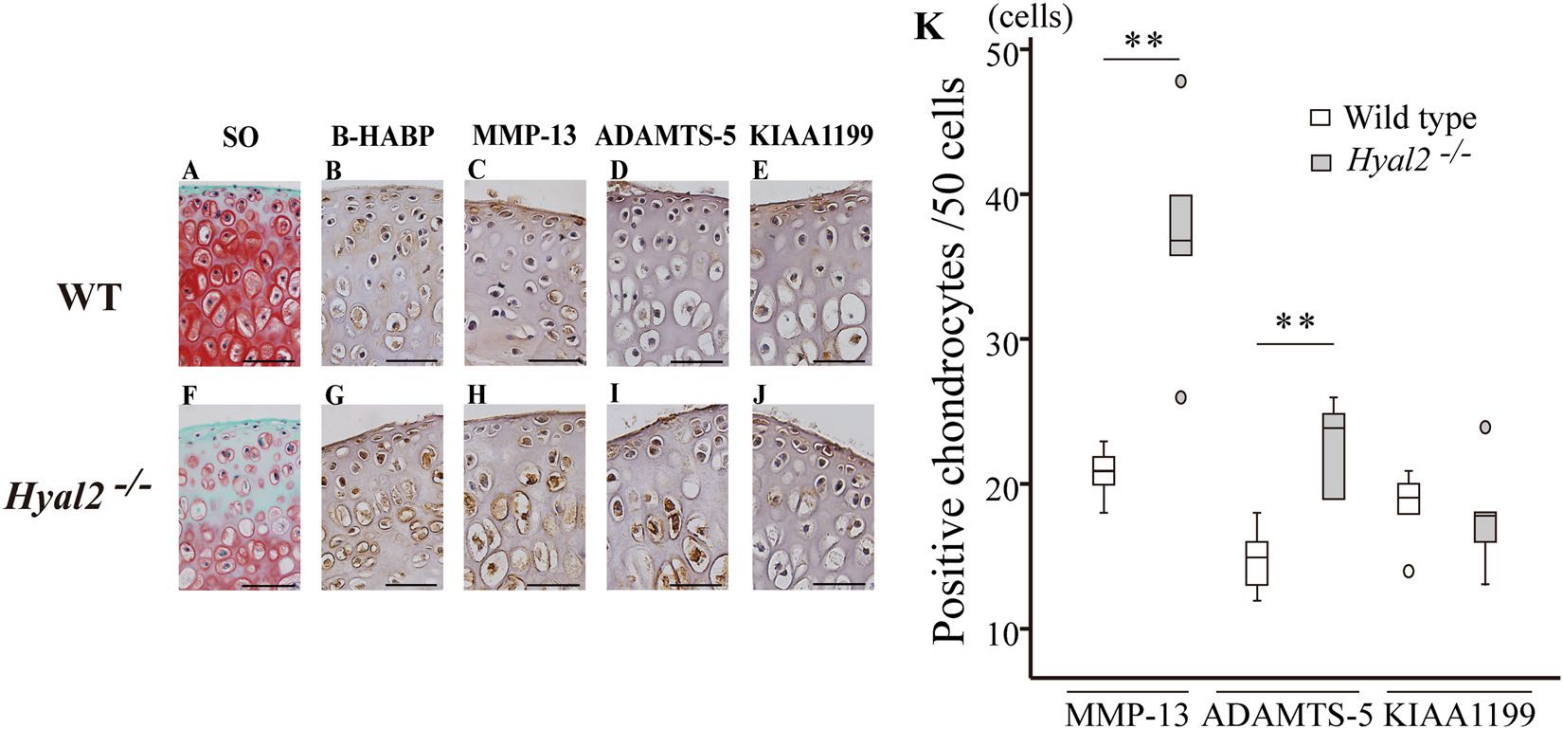
Articular cartilage explant culture with IL-1α of WT and *Hyal2* ^*−/−*^ mice. Pieces of articular cartilage was cultured for 72 h in serum free DMEM containing 10 ng/ml mouse IL-1α, and were subjected to Safranin O staining (SO) (A; WT, F; *Hyal2* ^*−/−*^), B-HABP staining (B; WT, G; *Hyal2* ^*−/−*^), MMP-13 staining (C; WT, H; *Hyal2* ^*−/−*^), ADAMTS-5 staining (D; WT, I; *Hyal2* ^*−/−*^), and KIAA1199 staining (E; WT, J; *Hyal2* ^*−/−*^). The numbers of positive stainable cells were graphed (** = P < 0.01) (K). Small circle represented outlier.

Three days’ culture with IL-1α resulted in a marked reduction of Safranin O positive staining in *Hyal2* ^*−/−*^ mice cartilage compared to that in WT mice cartilage (Fig. 7F and A, respectively). Results of immunohistochemistry showed that HA was abundantly deposited in and around the chondrocytes in *Hyal2* ^*−/−*^ mice (Fig. 7G) compared with WT mice (Fig. 7B). Dark positive staining with MMP-13 was observed in chondrocytes of *Hyal2* ^*−/−*^ cartilage (Fig. 7H), where stainable proteoglycan was reduced with safranin O staining, compared with that in WT cartilage (Fig. 7C). More MMP-13-positive chondrocytes were observed in *Hyal2* ^*−/−*^ articular cartilage (P < 0.01, Fig. 7K) Also, higher positive staining for ADAMTS-5 was observed in chondrocytes of *Hyal2* ^*−/−*^ cartilage (Fig. 7I) compared with those of WT (Fig. 7D). Staining pattern with anti-KIAA1199 antibody was similar in *Hyal2* ^*−/−*^ and WT cartilage (Fig. 7J and E, respectively). There was no significant difference in the ratio of articular chondrocytes positive for KIAA1199 between the two groups. (Fig. 7K).

## Discussion

The results of the present study indicated that *Hyal2* knockdown selectively in cartilage accelerates OA development not only in the natural course of aged mice, but also in a DMM induced OA model. Effects of *Hyal2* knockdown on neonatal or young mice knee joints were minimal, but were pronounced under conditions more prone to OA including increased age and in a DMM model.

A previous study investigating *Hyal1* knockout mice demonstrated that knee joints of the mice showed an increased positive pericellular and/or cytoplasmic HA staining and a reduction of proteoglycans at as early as 3 months of age, and showed progression at 12 months^16^. The OA change was observed earlier in their study compared with the present one, possibly because of differences in HA catabolic activity between Hyal1 and Hyal2. The enzymatic activity of Hyal2 is reported to be lower than that of Hyal1^5^. Another reason might be some difference in the cell population, which was *Hyal*-knocked down. In their study, another OA-related cell population was also *Hyal1*-knocked down, including synovial cells, macrophages, and osteoblasts/osteoclasts. Whereas, in the present study, *Hyal2* knockdown is Col2a1 promoter specific. One possibility would be that Hyal2 depletion does not cause marked osteoarthritic change in mice. We have preliminarily examined the expression levels of HYAL2 mRNA in human articular cartilage. Interestingly, in non-OA cartilage evaluated with HE and safranin-O staining, levels of HYAL2 mRNA differed among cases, suggesting that HYAL2 levels might not directly be associated with OA development (Supplementary protocol 1 and Supplementary Fig. S2). Because human and mouse articular chondrocytes express the three most broadly distributed HYAL1/Hyal1, HYAL2/Hyal2 and HYAL3/Hyal3^20, 21^, the roles of Hyal3 in the development of OA require investigation.

Atumuri *et al*. analyzed *Hyal3* knockout mice. *Hyal3* ^*−/−*^ mice were viable, fertile and exhibited no gross phenotypic abnormalities. Moreover, investigations of knee joint tissues from *Hyal3* ^*−/−*^ mice at 12–14 months of age exhibited no difference between the knee joint tissues from the *Hyal3* ^*−/−*^ and *Hyal3* ^*+/+*^ mice^17^. These findings suggest that Hyal3 does not have crucial roles in the extracellular matrix degradation of articular cartilage. A previous study established and analyzed *Hyal2* ^*−/−*^ mice using PGK-Cre system, and revealed that they exhibit localized congenital defects in frontonasal and vertebral bone formation^3^. Compared with the present study using Col2a1-Cre system, they used relatively young mice, and articular cartilage was not investigated. Moreover, a disease model such as DMM in our study was not used.

MMP-13 and ADAMTS-5 are thought to be one of the crucial enzymes responsible for collagen and aggrecan degradation in articular cartilage^22–25^. MMP-13 is thought to play very important roles in the degradation of collagen within the cartilage, due to its preferential digestion of type II collagen over types І and III^22, 24, 25^. In three different OA conditions (age-associated, instability-induced, and explant culture with IL-1α), *Hyal2* ^*−/−*^ mice displayed increased expression of MMP-13 and ADAMTS-5 when compared to WT mice. The mechanisms by which excessive storage of HA induces the catabolic cytokines are unknown. Simonaro *et al*. investigated the articular cartilage and cultured articular chondrocytes from rats and cats with MPS VI. In their study an age-related increase in the number of apoptotic chondrocytes was identified possibly due to the accumulation of dermatan sulfate, and articular chondrocytes from MPS VI animals released more nitric oxide and tumor necrosis factor alpha^26^. The accumulation of HA within the articular cartilage of the *Hyal2* ^*−/−*^ mice at 9 months, and DMM mice of *Hyal2* ^*−/−*^ might induce the secretion of inflammatory cytokines like in MPS VI models. Precise mechanisms should be analyzed further.

Recently, KIAA1199, a deafness gene of unknown function, has been shown to have HA degrading activity independent of CD44 and Hyal enzymes, particularly in dermal fibroblasts in normal skin and synovial cells in inflamed joints^27^. In the present study, the expression of KIAA1199 in the articular chondrocytes did not increase with OA progression, or in *Hyal2* ^*-/-*^ compared with WT mice. These findings suggest that HA degrading activity by Hyal enzymes and KIAA1199 acts differently.

OA change in subchondral bone has been reported as a two-stage process. A recent review has proposed that micro damage to the subchondral bone results in increased resorption and a decrease in the thickness in the early stage of OA. Subsequently subchondral trabecular sclerosis occurs and calcified cartilage thickness increases in the late stage of OA^28, 29^.

In our study, Hyal2 was expressed in growth plate cartilage, which is consistent with a previous report describing that both Hyal1 and Hyal2 mRNAs were highly expressed at the proliferative and hypertrophic layers of rabbit growth plate^21^. *Hyal2* knockout in growth plate cartilage resulted in the accumulation of HA. *Hyal2* deficiency has significantly caused the accumulation of hyaluronan in joint cartilage, high molecular weight HA content increases compared with WT mice.

Because a previous report indicated that high molecular weight hyaluronic acid (HMW-HA) prevented osteoclast differentiation and inhibited osteoclastogenesis^30^, HMW-HA, which accumulated in the growth plate cartilage because of *Hyal2* deficiency, might have affected subchondral bone formation. In fact, bone histomorphometric parameters of *Hyal2* ^*−/−*^ sham side at 10 weeks were lower compared to those of WT mice. However, at 9 months of age, the difference in bone histomorphometric parameters was reduced between *Hyal2* ^*−/−*^ and WT mice. These findings suggest that *Hyal2* deficiency in growth plate cartilage might affect epiphyseal bone formation just slightly.

It has been reported that *Hyal2* knockout mice do not alter HAS expression such as HAS1 and HAS2^31^, and these results were similar to the results of this study. Whereas, *Hyal2* knockout mice had higher Hyal1 expression in the kidneys than wild type mice^31^. In this study, there was no significant difference in mRNA expression of Hyal1 between *Hyal2* ^*−/−*^ and WT mice. This may have represented the difference of HA catabolism between cartilage and kidney.

This study has several limitations. First, the precise mechanism of OA development in Hyal2 deficient animals is not fully understood. As was observed in other MPS animals, which were characterized by accumulation of glycosaminoglycans (GAG), articular cartilage exhibits OA change^16^ and articular chondrocytes became apoptotic and release more inflammatory cytokines^26^. Second, we evaluated mRNA expression of MMP-13, ADAMTS-5 and KIAA1199, and the amounts and molecular sizes of HA in the cartilage of *Hyal2* ^*−/−*^ and WT mice. A tolerable level of error may be expected in these analyses because of the minimum amount of bone contamination. Furthermore, we analyzed the amounts and molecular sizes of HA in the cartilage of 9-month-old male *Hyal2* ^*−/−*^ and WT mice. The results might be influenced by osteoarthritic change of knee joints.

Other limitations include the lack of investigation as to why there was a difference in body weight at 10 weeks after DMM surgery between *Hyal2* ^*−/−*^ and WT mice. Although gait analyses of these mice were not performed in this study, progression of osteoarthritic change in knees might have exerted a negative influence on the locomotion of *Hyal2* ^*−/−*^ mice, leading to the increase noted in their body weight.

In conclusion, the current study demonstrated that the absence of Hyal2 in articular cartilage was associated with accumulation of HA and stimulation of OA development and progression. It was previously reported that Hyal1 deficiency in a mouse model of MPS IX exhibited OA. Hyal2 deficiency showed similar OA features, suggesting a novel MPS-like disorder of HA storage.

## Methods

### Mice

All animal experiments were performed in accordance with the Institute for Laboratory Animal Research, National Research Council of the National Academies Guide for the Care and Use of Laboratory Animals under the approval of the Institutional Animal Care and Use Committee at Nagoya University (approved number: 28068). FLP mice in C57BL/6 J background were provided by Unitech Co., Ltd (Chiba, Japan). Age matched male C57BL/6 J WT mice were used as a control. All mice were housed in a temperature and humidity controlled environment under 12 – h light / 12 h dark cycle and fed a standard rodent diet.

### Generation of cartilage specific conditional *Hyal2*^*−/−*^ mice

Design of the replacement vector (Supplementary Figure S3), Generation of the homologous recombination ES cells, Generation of *Hyal2* ^*+/Flox*^ mice, and Generation of Col2a1 promoter inducible conditionally *Hyal2* ^*−/−*^ mice were described in Supplementary protocol 2.

### Natural course and instability-induced OA model of *Hyal2*^*−/−*^ mice

Initially, to investigate the roles of Hyal2 in the development of OA in non-treated mice, knee joints of 3- and 9-month-old male WT and Col2a1 conditional *Hyal2* ^*−/−*^ mice were removed as specimens of the natural course^32^. We evaluated the macroscopic phenotype of WT and *Hyal2* ^*−/−*^ including body weight, and a soft radiograph of the skeleton was obtained using a Softex apparatus (Type EMB).

Destabilization of the medial meniscus (DMM) surgery was performed as previously described^33–35^ using 10-week-old male WT and *Hyal2* ^*−/−*^ mice. Briefly, mice were anesthetized with a 0.1 ml intraperitoneal injection of pentobarbital sodium (10 mg/ml). Under sterile conditions, the skin was incised followed by medial parapatellar arthrotomy, and the anterior fat pad was dissected to expose the anterior medial meniscotibial ligament, which was severed. A skin incision and medial parapatellar arthrotomy were performed in the left knees (sham side) of each mouse. *Hyal2* ^*−/−*^ (n = 24) and WT (n = 24) mice were subjected to this procedure. To investigate the time course of OA development after DMM or sham surgery, mice (n = 8 each) were sacrificed at 6, 8, 10 weeks after surgery, and the knee joints were excised and subjected to histologic analyses.

### Explant culture of femoral head cartilage

Since previous studies reported the use of IL-1α^36, 37^, to investigate the effects of this catabolic mediator on *Hyal2* deficient articular cartilage, femoral head cartilage (2 mm × 2 mm) of *Hyal2* ^*−/−*^ and WT mice was obtained, and subjected to explant cultures.

The femoral head cartilage was harvested from 4-week-old mice (n = 5) and pre-cultured for 48 h at 37 °C in a humidified atmosphere of 5% CO2 and 95% air, using Dulbecco’s modified Eagle’s medium (DMEM) containing 1% antibiotic solution (penicillin-streptomycin, Sigma-Aldrich), 2 mM glutamine, 10 mM 4-(2-HydroxyEthyl)−1-PiperazineEthaneSulfonicacid (HEPES), 50 µg/ml ascorbate (Sigma-Aldrich), and 10% fetal bovine serum (FBS). The explants were washed three times with serum free DMEM and cultured for an additional 72 h in serum free DMEM containing 10 ng/ml mouse IL-1α (Cell Signaling Technology, Danvers, MA, USA).

### Histologic evaluation of OA

Mice were trans-cardially perfused with cold 4% paraformaldehyde (PFA), and the knee joint was post-fixed in 4% PFA at 4 °C for 12 hours. After washing with phosphate buffered saline (PBS), the samples were decalcified in 10% EDTA solution at 4 °C for 3 weeks and then embedded in paraffin. Coronal sections (5 µm) were cut and mounted onto silicone coated slides.

OA change was analyzed in coronal sections of knee joints and graded according to a modified Mankin histologic score for the tibial articular side in all weight bearing areas of the femoro-tibial joint^22, 38^. Each sample was stained with Safranin O. Ten coronal sections around the midline of the medial tibia were examined in each mouse. A total modified Mankin score representing the overall state of the cartilage in the joint was summed and averaged from the scores of ten individual sections. This grade of OA was determined by 2 observers (EK and DM), who were blinded to the experimental details to avoid observer bias. Explant cultures of mice femoral head were also subjected to staining with HE or Safranin O to assess the presence of proteoglycan.

### Immunohistochemistry

Tissue sections adjacent to those evaluated with modified Mankin score and femoral head were subjected to immunohistochemical analyses according to the method previously described^22^. To unmask the extracellular matrix around the epitope, the sections were incubated with chondroitinase ABC (0.25 units/ml, pH 8.0, Sigma-Aldrich) overnight at 4 °C. Primary antibodies used were rabbit anti-Hyal2 polyclonal antibody (1:100 dilution; Abcam, Cambridge, MA, USA), mouse anti-MMP-13 polyclonal antibody (1:100 dilution; Proteintech, Chicago, IL, USA), rabbit anti-ADAMTS-5 polyclonal antibody (1:100 dilution; Abcam), rabbit anti-KIAA1199 polyclonal antibody (1:100 dilution; Proteintech), and biotinylated HABP (1:100 dilution; SEIKAGAKU BIOBUSINESS, Tokyo, Japan). All antibodies were confirmed to cross-react mice antigens as shown in our previous study^39^ and datasheet provided by manufacturers. After washing, sections were subjected to the usual staining procedures. For negative controls, tissue sections were incubated with IgG from non-immunized rabbit as a substitute for the first antibody.

The number of positively stained cells was counted in articular cartilage. Each section was observed under a light microscope at 400× magnification. The mean number of positive cells/50 cells was calculated from 8 mice in each group. All slides were evaluated independently by 2 blinded observers (EK and DM).

### Real-time reverse transcriptase-polymerase chain reaction (RT-PCR)

To evaluate the mRNA expression of HAS1, HAS2, HAS3, Hyal1, Hyal2, KIAA1199, MMP-13, and ADAMTS-5, cartilage samples from 9-month-old male *Hyal2* ^*−/−*^ and WT mice were obtained and subjected to the RT-PCR analyses according to a previous report^40^. Liver and triceps surae muscle from the *Hyal2* ^*−/−*^ and WT mice were retrieved and subjected to RT-PCR as a positive control for Hyal2 expression and to confirm the cartilage specificity of the Col2a1 promoter *Hyal2* ^*−/−*^ mice. The relative levels of mRNA in a sample were expressed after normalization with those of glyceraldehyde-3-phosphate dehydrogenase (Gapdh). Details of the primers used are listed in Supplementary Table S3.

### Gel filtration of cartilage glycosaminoglycan

Methods for the preparation of cartilage glycosaminoglycans is provided as Supplementary protocol 3. Aliquots of cartilage glycosaminoglycan preparations from *Hyal2* ^*−/−*^ or WT mice (n = 6, respectively) were pooled and applied on to an analytical Sephacryl S-1000 column (0.7 × 40 cm). The column was eluted with PBS solution at a flow rate of 0.5 ml/min, and 0.5 ml fractions were collected. Hyaluronan contents in each cartilage glycosaminoglycan preparation and the column fractions were determined using a competitive ELISA^18^. Hyaluronan references with known molecular masses (2130, 460, 150 and 5.6 kDa, a courtesy from Seikagaku Corp., Tokyo, Japan) were used to calibrate the column.

### Bone histomorphometry

To analyze the trabecular and cortical structure of the proximal tibia (epiphyses) in the natural course of *Hyal2* ^*−/−*^ and WT mice, and DMM *Hyal2* ^*−/−*^ and WT mice, at first, knee joints were scanned using the Skyscan 1072 micro-CT scanner (Skyscan, Kontich, Belgium) with 5 µm/pixel and reconstructed with software (NRecon software, SkyScan). To evaluate the trabecular and subchondral bone of the epiphyses, the epiphysis of the medial tibia was chosen as the region of interest. The target region in the epiphysis was selected using 3D data analysis software (CTA anlyser, Skyscan). The thickness of the medial side of the subchondral bone plate and trabecular bone was measured. An area (0.5 mm mediolateral width, 1.0 mm ventro-dorsal length) of the weight bearing region of the medial tibial plateau was taken as the region of interest^19^, and 394 slices of 9 µm thickness were reconstructed.

Bone morphometric parameters of targeted region in the tibial bone were determined using the freely available software package, 3D-Calculator. The indices examined included trabecular bone volume ratio (Tb BV/TV), trabecular thickness (TbTh), trabecular separation (TbSp), trabecular number (TbN), the bone volume ratio of subchondral plate (Sb BV/TV) and subchondral plate thickness (SbTh). These parameters were determined as previously described^41^.

### Statistical analysis

All data are expressed as the mean ± SD. Group means were compared using Student’s t-test or Mann-Whitney U test. Analysis of variance followed by Bonferroni correction or Dunn’s post hoc test was used to assess differences among multiple groups. These statistical analyses were performed using SPSS version 21 (IBM Corp., Armonk, NY). A p value < 0.05 was considered statistically significant.

### Data Availability

All data generated or analyzed during this study are included in this published article (and its Supplementary Information files. Associated protocols and data of this study are available for readers. Knockout mice could be available with material transfer agreement in our institution.

## Electronic supplementary material

Supplementary information
