# Supplementary material for: Conditional knockdown of hyaluronidase 2 in articular cartilage stimulates osteoarthritic progression in a mice model
Source: Sci Rep. 2017 Aug 1;7:7028. doi: 10.1038/s41598-017-07376-5 (PMC5539311; doi:10.1038/s41598-017-07376-5)
Supplement: Supplementary file 1 — Supplementary information [file 41598_2017_7376_MOESM1_ESM.pdf]

Title: Conditional knockdown of hyaluronidase 2 in articular cartilage stimulates osteoarthritic progression in a mice model

Authors:
Yoshitoshi. Higuchi, Yoshihiro Nishida, Eiji Kozawa, Lisheng Zhuo, Eisuke Arai, Shunsuke Hamada, Daigo Morita, Kunihiro Ikuta, Koji Kimata, Takahiro Ushida, Naoki Ishiguro

Supplementary Table S1.

Micro CT analysis at 9 months of age in mice with aging-associated OA

Parameter	WT<br>Lt<br>(n=7)	*Hyal2*$^{-/-}$<br>Lt<br>(n=7)
Sb BV/TV (%)	39.2 ± 11.3	34.2 ± 9.9
Sb Th (μm)	53.6 ± 11.1	47.2 ± 9.1
Tb BV/TV (%)	35.7 ± 4.3	35.0 ± 8.1
Tb Th (μm)	50.6 ± 2.8	46.9 ± 5.1
Tb Sp (μm)	97.9 ± 12.4	90.1 ± 15.3
Tb.N (/mm)	7.1 ± 0.7	7.7 ± 1.3

Supplementary Table S2.

Micro CT analysis at 6 weeks after DMM surgery

Parameter	WT DMM (n=8)	WT Sham (n=8)	*Hyal2*$^{-/-}$ DMM (n=8)	*Hyal2*$^{-/-}$ Sham (n=8)
Sb BV/TV (%)	50.7 ± 14.3	41.8 ± 15.2	43.0 ± 3.9	39.6 ± 6.1
Sb Th (µm)	53.3 ± 4.5	46.1 ± 10.4	46.5 ± 6.5	43.9 ± 4.9
Tb BV/TV (%)	37.9 ± 7.3	39.8 ± 8.1	32.1 ± 5.2	34.4 ± 10.8
Tb Th (µm)	60.0 ± 6.6	61.1 ± 5.5	54.1 ± 5.2	57.2 ± 3.5
Tb Sp (µm)	109.6 ± 21.0	96.7 ± 17.7	110.5 ± 13.9	101.3 ± 11.7
Tb.N (/mm)	6.6 ± 1.2	6.5 ± 1.3	6.3 ± 0.6	5.9 ± 1.7

Supplementary Table S3. Primer sequences

Gene		Primer sequence
MMP-13	Forward	5’- AGTTGACAGGCTCCGAGAAA -3’
	Reverse	5’- GGCACTCCACATCTTGGTTT -3’
ADAMTS-5	Forward	5’- CTTTAGAGGGAGAAAATTCTGG -3’
	Reverse	5’- AAAGATTTACCATTGGGTGG -3’
Hyal1	Forward	5’- CAAGTACCAAGGAATCATGCCAG -3’
	Reverse	5’- GCGGACACAGCGACCATG -3’
Hyal2	Forward	5’-TGTGGCTCTCACCTGGACCTTATGA -3’
	Reverse	5’-AGATGGTATGGGTGCTCTGCTAAG -3’
KIAA1199	Forward	5’- ATATACAGGCCACAACAATG -3’
	Reverse	5’- AAGCAAACCTGTAATCTTGG -3’
HAS1	Forward	5’- TCCTCTGGGTCTATACAGAAACAATC -3’
	Reverse	5’- CGGTTGGTGAGGTGCCTGT -3’
HAS2	Forward	5’- GATTATGTACAGGTGTGTGAC -3’
	Reverse	5’- CCTCTAAGACCTTCACCATC -3’
HAS3	Forward	5’- GATGTCCAAATCCTCAACAAG -3’
	Reverse	5’- CAAAGGCCCACTAATACATTG -3’
Gapdh	Forward	5’- ACCCAGAAGACTGTGGATGG -3’
	Reverse	5’- CACATTGGGGGTAGGAACAC -3’

Supplementary figure S1

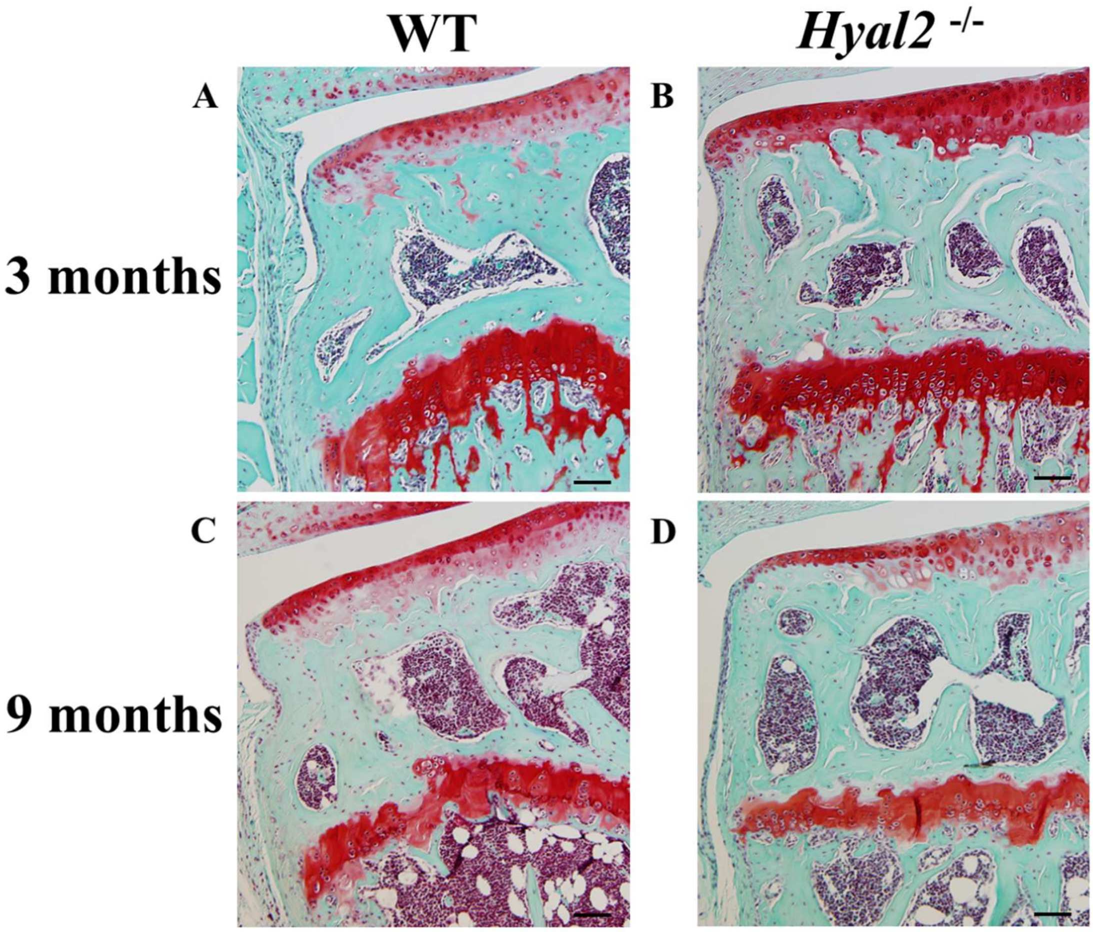

Supplementary figure S2

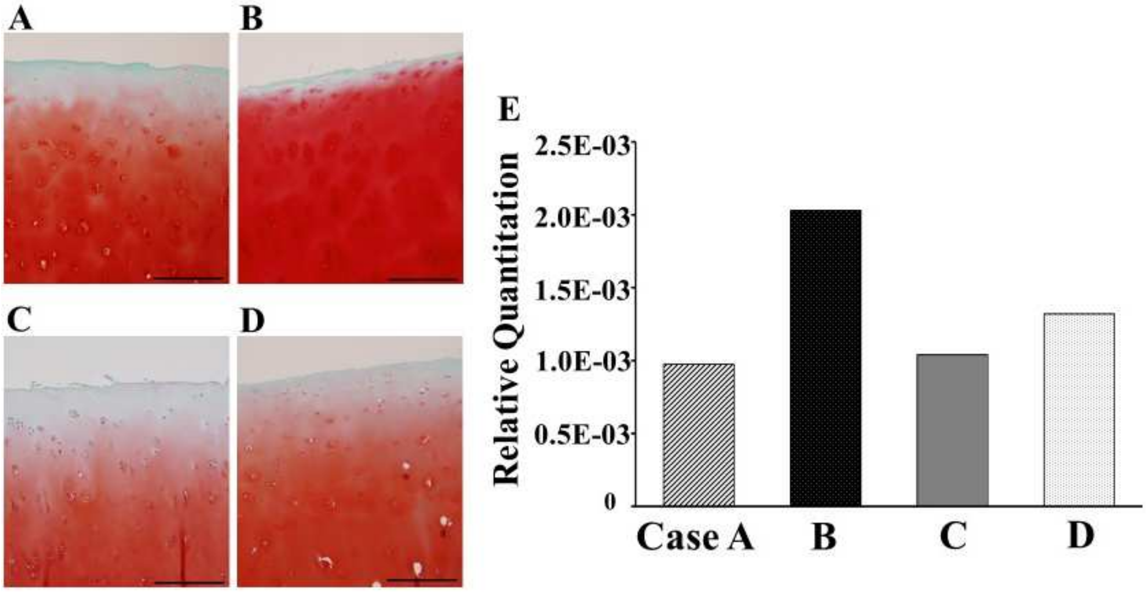

Supplementary figure S3

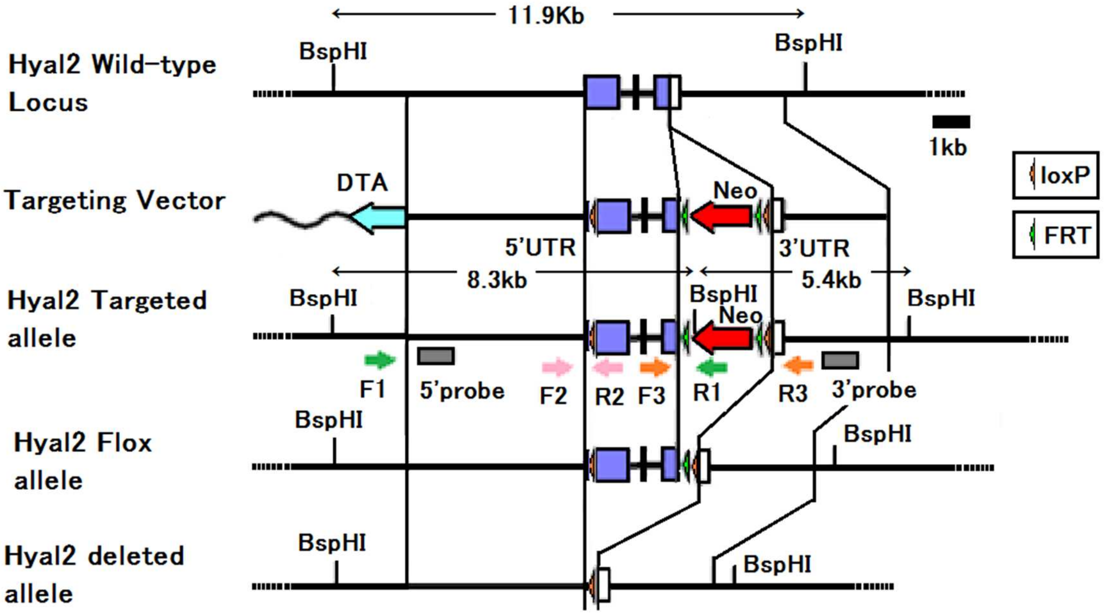

Supplementary Figure legends.

Supplementary Figure S1. Safranin O staining of growth plate and articular cartilage. Knee joints of WT (A and C) and *Hyal2*$^{-/-}$ (B and D) mice at 3 months (A and B) and 9 months of age (C and D). Growth plate may still be open in both groups at 3 months. The growth plate of both groups was thinner at 9 months compared to that at 3 months.

Supplementary Figure S2. HYAL2 mRNA expression in Safranin O well stained human articular cartilage

Macroscopically normal human cartilages were subjected to SO staining, and RT-PCR analyses for evaluation of HYAL2 mRNA expression. Four different cases were shown (A-D). HYAL2 mRNA expression levels were graphed after normalization with those of GAPDH (E). Scale bars depict 200 μm in length.

Supplementary Figure S3. Design of the replacement vector.

A fragment of C57BL/6J mouse genomic DNA containing Hyal2 was obtained from a BAC clone (clone ID : RP23-418E8 , Roswell Park Cancer Institute). Through successive subcloning steps, a loxP site was inserted in 5'UTR (untranslated region)

present in the exon which contains the ATG initiation codon of Hyal2, and the fragments were cloned into the FRT-neo-FRT-loxP-DTA targeting vector (Unitech Co., Ltd.) so that the coding region of Hyal2 gene is flanked by two loxP sites. Neomycin resistance cassette (FRT-neo-FRT) was the gene used as a selection mark flanked with two FRT sites. This resulted in floxing of the complete coding sequence of Hyal2 since exon 1 is noncoding. The appearance of the BspHI restriction sites present in the FRT site and the selection marker was used for identification of the targeted allele in subsequent Southern blot analyses.

Supplementary Protocol 1

Real-time reverse transcriptase-polymerase chain reaction (RT-PCR) for human HYAL2 and histologic evaluation

To evaluate the expression levels of HYAL2 mRNA in the human articular cartilage, four human macroscopically normal cartilages were obtained during total hip arthroplasty for the osteonecrosis of femoral head (n=4). The median age was 51.5 years (27-63 years). Obtained cartilage tissues were formaldehyde fixed and embedded in paraffin. The 5 μm thick sections were subjected to HE and Safranin O staining. To evaluate the mRNA expression of HYAL2, cartilage samples were subjected to the RT-PCR analyses. The expression levels of mRNA in a sample were expressed after normalization with those of glyceraldehyde-3-phosphate dehydrogenase (GAPDH). Following forward and reverse primer pairs were used: for *HYAL2*, 5’-AGTCAAACAGGCACAATATG-3’ (forward) and 5’-GATTGTAGCAGTCAGGAAAG-3’ (reverse); for *GAPDH*, 5’-TGCACCACCAACTGCTTAGC-3’ (forward) and 5’-GGCATGGACTGTGGTCATGAG -3’ (reverse).

Supplementary Protocol 2

Generation of the homologous recombination ES cells

The replacement vector was linearized by NotI site and electroporated into C57BL/6J embryonic stem (ES) cells that were cultured on G418-resistant mouse embryonic fibroblasts. The resistant colonies were picked up and screened for homologous recombination by polymerase chain reaction (PCR) using the R1 primer : 5’-CTTCCTCGTGCTTTACGGTATC-3’ (Neo-cassette specific primer) and the F1 primer : 5’-AAAGCCTACATCAGTATGTCAACG-3’. Correctly targeted clones were identified by standard Southern blot analysis using 5' and 3' probes external to the regions of homology and following BspHI digestion. In addition a Neo probe was used to screen for random insertion events. The 5'-probe was PCR-amplified using 5'-GCTATACTTGAACAACAGACACTTCC-3' and 5'-TGGTAGTGGTGAGATAGGATACTGC -3' as primers. The 3'-probe was PCR-amplified using 5'-CCTGTGACTCATTCTGTTGTAACC-3' and 5'-GTTTTATGTGCCTTGTTTAACCTTC-3' as primers. The Neo probe was PCR-amplified using 5'- GAACAAGATGGATTGCACGCAGGTTCTCCG -3' and 5'-CGCCAAGCTCTTCAGCAATA -3' as primers. Correctly targeted clone was obtained.

Generation of *Hyal2*$^{+/Flox}$ mice

Chimeras were generated by injecting the correctly targeted ES cells into blastocysts of Balb/c mice and identified by coat color. Chimeric male founder mice were mated to C57BL/6J female mice to generate F1 heterozygous mice for Hyal2^{Flox} line. F1 heterozygous mice were crossed with FLP mice to delete the neomycin cassette flanked

by two FRT sites. The loxP check was performed to confirm the insertion of the loxP, and PCR-amplified using the F2 primer : 5’-CATCTCTGGAATTTGCTAGACTGAC-3’ and the R2 primer : 5’-CCAGTGTGATGATGGGACCTAGT-3’. These mice were identified by PCR using the primer on the F3 primer : 5’- TGTGGCTCTCACCTGGACCTTATGA-3’ and the R3 primer : 5’- CCAGTGTGATGATGGGACCTAGT-3’.

Generation of Col2a1 promoter inducible conditionally *Hyal2*$^{-/-}$ mice

The offspring carrying the *Hyal2*$^{+/Flox}$ mice were bred with Col2a-Cre mice in order to achieve complete recombination to generate the conditional *Hyal2*$^{-/-}$ mice. Because preliminary experiment revealed that there was no difference in body weight and no findings of OA development in knees until 9 months of age in *Hyal2*$^{+/Flox}$, *Hyal2*$^{+/-}$, and C57BL/6J background mice, we used C57BL/6J mice as WT (wild type) control in this study. Genotypes were determined using PCR analysis. For PCR analysis, DNA isolated from ear-punches was PCR-amplified with specific Hyal2 primers to evaluate allele status. We used forward and reverse primers (5’-TGTGGCTCTCACCTGGACCTTATGA -3’) and (5’-AGATGGTATGGGTGCTCTGCTAAG -3’), respectively. The Hyal2 targeted and wild-type alleles exhibited PCR products of 318 bp and 164 bp bands, respectively. Genotypes of mice were identified as *Hyal2* (+/+), (-/-), or (+/-) by the presence of the 164 bp band only, 318 bp only, or both 318 and 164 bp bands, respectively.

Supplementary Protocol 3

Preparation of cartilage glycosaminoglycan

To analyze the amount and molecular sizes of HA in the cartilage of *Hyal2*$^{-/-}$ and WT mice, four joints included two knee joints and two femoral heads of 9-month-old male WT and *Hyal2*$^{-/-}$ mice were excised. Meniscus, joint capsule and bone were removed as much as possible. The four joints from each mice were placed in a test tube, frozen in liquid nitrogen, crushed to power with μT-12 beads crusher (TAITEC Corp., Saitama, Japan) following manufacturer's instruction. The powder in a test tube was suspended in 600 μl of 0.2N NaOH and then incubated overnight at room temperature. After neutralization with 1N HCl, the solutions were adjusted to contain 0.15M Tris, 0.15M NaCl, 0.01M CaCl2, 5mM deferoxamine mesylate and 20U/ml protease E at pH8, and further incubated at 55°C for 8 hours. After inactivation of protease E by heating at 95°C for 15 minutes, the solutions were centrifuged at 12,000 rpm for 10 minutes to remove insoluble materials. The supernatants were mixed with 3 volumes of 95% ethanol containing 1.3% of potassium acetate, incubated at -20°C for 30 minutes, and then centrifuged at 12,000 rpm for 10 minutes at 4°C. The pellets were washed once with cold 70% ethanol and then dissolved in PBS for further analysis.
